# Response to Letter to Editor by A. Derbalah et al.: the role of automation in enhancing reproducibility and interoperability of PBPK models

**DOI:** 10.1093/bib/bbaf060

**Published:** 2025-02-10

**Authors:** Elena Domínguez-Romero, Stanislav Mazurenko, Martin Scheringer, Vítor A P Martins dos Santos, Chris T Evelo, Mihail Anton, John M Hancock, Anže Županič, Maria Suarez-Diez

**Affiliations:** Department of Bioinformatics—BiGCaT, School of Nutrition and Translational Research in Metabolism, Faculty of Health, Medicine and Life Sciences, Maastricht University, P.O. Box 616, 6200 MD Maastricht, The Netherlands; RECETOX, Faculty of Science, Masaryk University, Kotlarska 2, 611 37 Brno, Czech Republic; Loschmidt Laboratories, Department of Experimental Biology and RECETOX, Faculty of Science, Masaryk University, Kamenice 5, 625 00 Brno, Czech Republic; International Clinical Research Center, St. Anne’s University Hospital Brno, Pekařská 53, 656 91 Brno, Czech Republic; RECETOX, Faculty of Science, Masaryk University, Kotlarska 2, 611 37 Brno, Czech Republic; Laboratory of Bioprocess Engineering, Wageningen University and Research, Droevendaalsesteeg 1, 6708 PB Wageningen, The Netherlands; Department of Bioinformatics—BiGCaT, School of Nutrition and Translational Research in Metabolism, Faculty of Health, Medicine and Life Sciences, Maastricht University, P.O. Box 616, 6200 MD Maastricht, The Netherlands; ELIXIR, Wellcome Genome Campus, Hinxton, Cambridgeshire CB10 1SD, United Kingdom; Department of Life Sciences, National Bioinformatics Infrastructure Sweden (NBIS), SciLifeLab, Chalmers University of Technology, Kemigården 1, Gothenburg SE-412 96, Sweden; Institute of Biochemistry and Molecular Genetics, Faculty of Medicine, University of Ljubljana, Zaloška 4, 1000 Ljubljana, Slovenia; Department of Biotechnology and Systems Biology, National Institute of Biology, Večna pot 121, 1000 Ljubljana, Slovenia; Laboratory of Systems and Synthetic Biology (SSB), Wageningen University and Research, Agrotechnology and Food Sciences, Stippeneng 4, 6708 WE Wageningen, The Netherlands

**Keywords:** reproducibility, PBPK models, standardization, automation

Dear colleagues,

We thank you for your comments on our manuscript [1] and for raising the important question of automation [2].

The publications by Sepp *et al.* (2019) [3] and Liu *et al.* (2024) [4] focus on one type of PBPK models, denominated ‘for biologics’. In these models, each compartment (tissue/organ) is divided into vascular, endothelial endosomal and interstitial (sub-)compartments, and lymphatic flows are also represented [3, 4]. In these examples, the pharmacokinetics of proteins are represented. Notably, the protein distribution within tissues includes processes of passive transport across two types of pores via diffusion or fluid convection, processes of pinocytosis, binding, recycling and degradation [4]. This type of PBPK models is complex and their authors successfully used specific tools for automated code generation. In particular, Liu *et al.* (2024) used ‘mathematical sets’ available in the *Ubiquity* package, which facilitates assembling model components in R-language [4]. Sepp *et al.* (2019) used the MATLAB script *PBPKassembler.m* for automated code generation, where files in Simbiology (MATLAB) and Excel formats were combined [3].

Briefly, automation can help build models, which is highly valuable notably for complex models. Having a robust, thoroughly validated platform for automated code generation will indeed contribute to accessibility and reproducibility. Also, automation of model building seems to be a natural way to address the case of large models, in which manual scripting will be prone to introducing errors.

Smaller models or those with fewer equations do not necessarily require automation. From our experience, writing the script manually provides a deep understanding of the underlying model mechanics. Manually written scripts also allow to give a clear view of how equations and parameters are applied, which we consider especially valuable for teaching and knowledge transfer.

To conclude, as observed in other niches of systems biology, automation brings significant benefits to the modelling field, e.g. there is a plethora of tools that reconstruct and benchmark genome-scale metabolic models. As the correspondence authors argue, there is more scope for automation in the current practices of PBPK modelling. To facilitate this, we have opted to begin the journey of standardization via open collaboration through ELIXIR. We are looking forward to continuing to engage the research community towards automatic construction, validation and deposition of PBPK models.

Thank you. Best regards,

Elena Domínguez-Romero, Stanislav Mazurenko, Martin Scheringer, Vítor Martins dos Santos, Chris Evelo, Mihail Anton, John M. Hancock, Anže Županič, and Maria Suarez-Diez.
